# Additive manufacturing of 3D flow-focusing millifluidics for the production of curable microdroplets

**DOI:** 10.1039/d4ra07234k

**Published:** 2024-12-12

**Authors:** Muhammad Saeed Saleem, Timothy T. K. Chan, Michel Versluis, Dominik Krug, Guillaume Lajoinie

**Affiliations:** a Physics of Fluids Group, Max Planck University of Twente Center for Complex Fluid Dynamics, University of Twente P.O. Box 217 7500 AE Enschede The Netherlands g.p.r.lajoinie@utwente.nl; b J. M. Burgers Centre for Fluids Dynamics, University of Twente P.O. Box 217 7500 AE Enschede The Netherlands; c Institute of Aerodynamics, RWTH Aachen University Wüllnerstraße 5a 52062 Aachen Germany

## Abstract

Microfluidics plays a crucial role in the generation of mono-sized microdroplet emulsions. Traditional glass microfluidic chips typically lack versatility in generating curable droplets of arbitrary liquids due to the inherent hydrophilic nature of glass and to fabrication constraints. To overcome this, we designed a microdroplet generator with 3D flow-focusing capabilities that can be 3D-printed. The chip can handle oil-in-water emulsions despite its lipophilicity. Operating in the jetting regime, the chip exploits the Rayleigh–Plateau instability to enable high throughput. With its versatile design, the chip is capable of producing both single and double emulsions within the same channel. We utilize a thermoset (epoxy–melamine) based system to test its ability to handle curable chemicals and to produce in a post-processing step both solid particles and filled capsules. With a low solvent concentration in the curable material, the present system can encapsulate water-based cores of a wide range of sizes.

## Abstract 2

3D flow-focusing for the production complex polymeric capsules.

## Graphical abstract

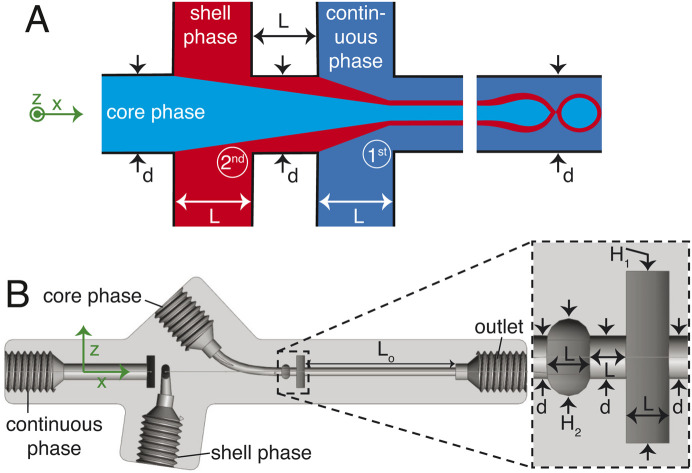

## 1 Introduction

Monodisperse particles and capsules hold high technological value due to their broad range of applications from fundamental scientific research to industry. Neutrally buoyant particles can be used as tracers to visualize flows,^1,2^ heavy particles can be employed as model particles to improve our understanding of how mineral extraction from an aqueous slurry of the ground ore.^3^ The use of microcapsules spans across pharmaceuticals,^4,5^ enabling the precise and localized release of drugs, agriculture^6,7^ for slow-release micro-encapsulated fertilizer, and energy storage as a phase change material.^8^ Producing particles and capsules with fine-tuned properties is therefore both scientifically appealing and technologically important.

The most common methods to produce small monodispersed particles is by curing droplets.^9–13^ The first step in this approach is the generation of droplets. Droplets are typically generated in microsieves,^14,15^ where the liquid breaks up into droplets after passing through an orifice, or microfluidic chips with coaxial flow, a T-junction, or flow-focusing geometries.^16,17^ The flow-focusing geometry as utilized by Yobas *et al.* (2006)^18^ to produce water-in-oil and oil-in-water droplets is especially interesting as it can reach high production rates of 10 000 Hz and 1000 Hz, respectively. In this geometry, a jet formed by the dispersed phase is squeezed from two sides by the surrounding liquid (continuous phase) to form droplets.

One of the challenges in the design of microfluidic droplet generators is to ensure that the channels can effectively handle both water/oil and oil/water-based systems with excellent wetting properties. Glass, which is widely employed for microfluidic chips due to its excellent durability and optical transparency, is hydrophilic and typically manufactured with 2D (planar) geometries. When water is used as the dispersed phase, the jet can adhere to the upper/bottom walls, hindering droplet formation. In this case, surface treatments are necessary to increase hydrophobicity of the chip while introducing additional variables in the chip design process.

In principle, the wetting effects can be eliminated by employing three-dimensional flow geometries where the jet is axisymmetrically contained by the continuous phase. While these geometries are complex and expensive to fabricate in glass chips, it is applicable in rapid-prototyping types of chips with polydimethylsiloxane (PDMS). This approach was effectively implemented with PDMS, as demonstrated by Castro-Hernández *et al.* (2016),^17^ where a 3D chip was developed to produce water-in-oil droplets. Recent advancements in PDMS-based microfluidics are thoroughly discussed in the work of Raj and Chakraborty (2020).^19^ However, this method requires the fabrication of a silicon mask in a clean room, followed by a time-consuming and labor-intensive process to form the necessary channels.

Manufacturing microfluidic droplet generators through 3D-printing is an attractive alternative as it offers much greater freedom in the chip geometry, greatly simplifies the manufacturing process and reduces the cost when produced on a small scale. Various studies (see *e.g.* ref. 20 and 21) have reported droplet generators with a range of geometries and liquids. One of the first attempts was performed by Shallan *et al.* (2014),^22^ who qualitatively demonstrated the concept by generating aqueous droplets in an organic solvent. Studies followed which employed chips with various geometries (with rectangular or circular channel cross-sections) and either the flow-focusing or T-junction configuration, see *e.g.* ref. 23 and 24. Bhargava *et al.* (2014)^23^ produced water-in-oil droplets by operating a flow-focusing chip in the jetting regime. Donvito *et al.* (2015)^24^ and Dewandre *et al.* (2020)^25^ showed that the monodispersity of droplets generated using 3D-printed chips is comparable to conventionally manufactured devices. Building on these successes, an introductory extension to more complex droplet morphologies, such as double emulsions,^10^ has been achieved by linking multiple droplet generators in series using O-rings.^26^

Producing droplets is only the first part of the process of making particles or capsules since the liquid droplets still need to be solidified. Solvent evaporation,^27^ utilized *e.g.* by Visscher *et al.* (2019),^15^ can be used to cure droplets. In this process, liquid droplets containing either dichloromethane, hexadecane, and PMMA or dichloromethane, perfluorocarbon oils, and PGLA can be generated. By stirring the solution, the droplets remain suspended, giving time for the solvent to evaporate yielding particles or capsules. Particles have also been produced by utilizing droplets consisting of photo-active^10,28^ and heat-activated resins.^12^ Despite the advantages of 3D-printed microfluidic chips, only recently Zhang *et al.* (2023)^12^ utilized them to produce curable droplets with complex chemicals.

In this work, we make use of the versatility and cost-effectiveness of 3D-printing to design a fully three-dimensional flow-focusing millifluidics channel that can produce curable single- and double-emulsion microdroplets. The chip is operated in the jetting regime for high production rates. Despite the inherent lipophilicity of the chip material, the present chip geometry does not require any surface treatment to produce droplets with an oil-based dispersed phase. We employ a model system based on a thermoset resin comprising epoxy–melamine to demonstrate the chip's ability to handle complex curable mixtures. Following droplet production, single-emulsion droplets are cured into particles by stirring and heating, while the double-emulsion droplets undergo shell polymerization to form capsules.

## 2 Materials and methods

### 2.1 3D millifluidic chip

**Fig. 1:**
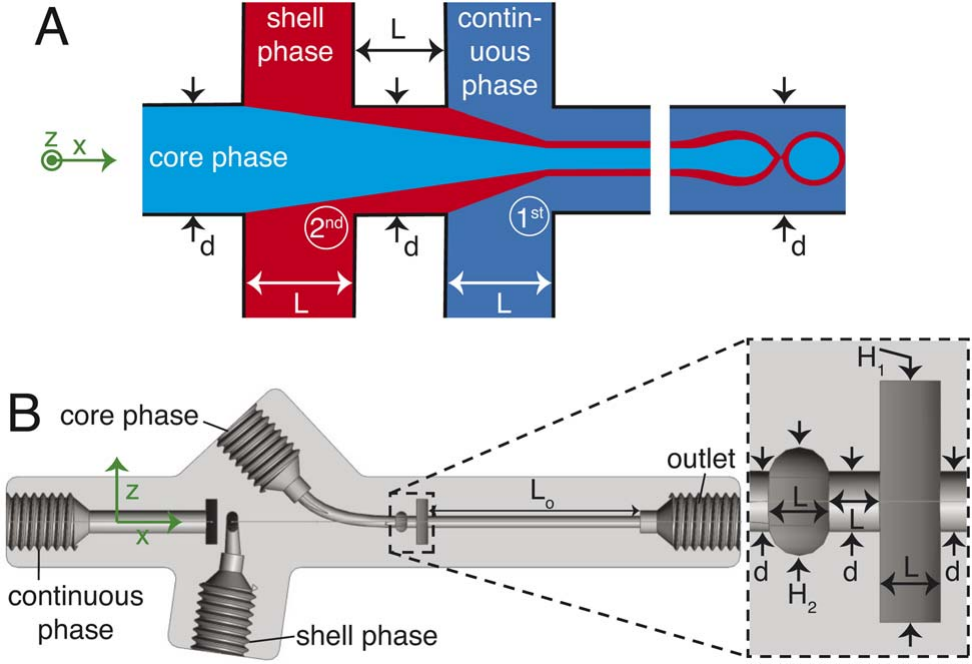
(A) A sketch illustrating a double emulsion flow junction. (B) A SolidWorks® cross-section view of the droplet generator along the centreline. *H*_1_ = 4 mm, *H*_2_ = 2 mm, *d* = *L* = 1 mm, *L*_o_ = 19 mm. The coordinate system is shown in green.

The chip is printed using a Formlabs Form 3+ 3D printer with clear v4 resin. Following printing, the chip is cleaned thoroughly with isopropyl alcohol (IPA), air-dried, coated on one side with the same resin for improved optical transparency, and subsequently cured at 60 °C for 7 hours. A sketch of the 3D millifluidic chip demonstrating the formation of double emulsion droplets is shown in Fig. 1A. To form capsules that encapsulate pure water, a water-based continuous phase and an oil-based shell phase are formulated and will be discussed later in Section 2.2. The chip comprises two flow-focusing junctions and four channels: one for a continuous phase, two for the dispersed phases (shell and core), and an outlet channel where droplets are collected. At the first junction, the shell phase is focused by the continuous phase while at the second junction, the core phase is focused by the shell phase. Downstream in the outlet channel, a co-flow is generated where the jet breaks up into droplets due to the Rayleigh–Plateau instability.

A cross-section of the 3D millifluidic nozzle design is shown in Fig. 1B. At both junctions, the channel height of the focusing phase is always higher than the phase being focused. This allows non-planar flow-focusing and prevents the jet from wetting the channel walls. This concept was applied by Castro-Hernández *et al.* (2016)^17^ to generate micron-size droplets with a junction height of 100 μm. In the present case, the first junction has a height (*H*_1_) of 4 mm and the second junction has a height of 2 mm. We observed that the 3D-printed material has an inherent lipophilic affinity. Therefore wetting from the oil-based shell phase is prevented by keeping the height of the first junction at 4 mm thereby dissipating surface energy, removing a need for surface treatment. The length of the outlet channel (*L*_o_) is kept at 19 mm to ensure sufficient space for jet formation and breakup.

### 2.2 Continuous and dispersed phases

The continuous phase is comprised of 40% glycerol and 60% water. This optimum mixture was selected based on a systematic study where the glycerol concentration was varied from 5% to 74%. At low concentrations, the coaxial jet oscillates laterally in the channel, whereas at high concentrations, the droplets coalesce in the outlet tube resulting in slug flow. For the shell phase, we selected a polymer mixture consisting of 1 g (1.17 g ml^−1^) of epoxy resin, 1 g (1.163 g ml^−1^) of hardener, and 0.5 ml melamine crosslinker, with 0.2 g 0.98 g ml^−1^ of dinonylnaphthalenedisulfonic acid (DNNDSA) acting as a catalyst. The acid's hydrophobic nature inhibits diffusion of the curing resins into the surrounding liquid. Upon addition of 0.1 mg of Nile red dye the mixture had a total volume of *V*_diss_ ≈ 2.42 ml of which 10% is butanol (BU). The mixture is dissolved in dichloromethane whose concentration varies for particles and capsules as will be discussed in Section 3. The optimal concentration of each component was determined (except DCM) *via* trial and error by mixing them in a Petri dish and subsequently heating the mixture to 40 °C in an oven, aiming to achieve the minimal reaction time to gel *i.e.* losing fluidity. The core phase for double-emulsion droplets is composed of water. After the droplets are produced, they will be heated to accelerate solvent evaporation and initiate crosslinking. We anticipate that these processes will occur simultaneously, as both are heat accelerated. This simultaneous action is expected to stabilize the double-emulsion droplets into a core–shell configuration during solidification. The detailed production and curing process is discussed in Section 2.3.

### 2.3 Operation

#### 2.3.1 Production process

**Fig. 2:**
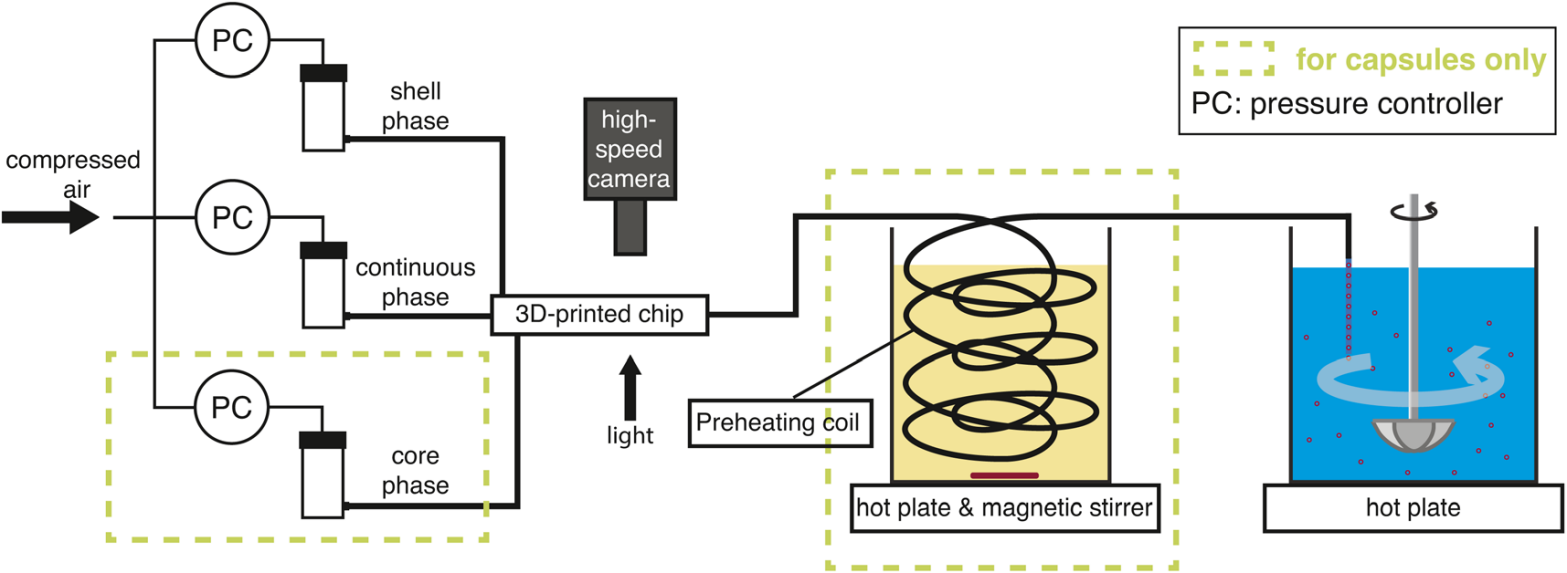
Schematic of the experimental setup. The chemicals are forced into the fluidic chip with compressed air to form droplets, which are subsequently cured into particles or capsules by stirring and heating.

Fig. 2 displays a schematic of the setup for producing microparticles or microcapsules. To generate single emulsion droplets, the core phase inlet is initially left open, and the continuous phase is first allowed to wet the channels. As the continuous phase begins to drip from the outlet, the core phase channel is sealed with a fingertight microfluidic plug. The shell phase is then slowly introduced to form a jet that breaks up into droplets. Since there is no shell present in the single emulsion, the shell phase hereafter is referred to as the dispersed phase. In the case of double emulsion, the core phase is also infused next to the shell phase creating a coaxial jet that breaks up into double-emulsion droplets. All the connections on the chip were made with PEEK fingertight connectors and PEEK tubing with an inner diameter of 1 mm except for the core phase, where 0.5 mm tubing is used in view of the lower liquid viscosity. The entire process is observed using a high-speed camera (Photron Fastcam SA-X2) connected to a Navitar 12× adjustable zoom lens with back illumination.

#### 2.3.2 Curing process

After the droplets are produced, the suspension passes through an outlet tube. For the double-emulsion case, the outlet tube is connected to a coil immersed in a 50 °C sunflower-oil-bath to trigger polymer network formation in the shell. The bath was stirred by a magnetic stirrer at 900 rpm to reduce thermal gradients. Once the droplets exit the outlet tube, they are collected in a beaker filled with 5% Tween 80-water solution at 40 °C and cured into particles and capsules. The mixture is stirred using a Heidolph overhead stirrer at 150 rpm during collection and at 250 rpm for the remainder of the process to prevent sedimentation. Over the next 4 hours, the solution is maintained at 40 °C for the particles or capsules to reach maximum polymer strength. This elevated temperature, which is the solvent's boiling point, triggers fast evaporation^29^ and is therefore expected to quickly stabilize the curing droplets, especially for the double-emulsion droplets during the initial stage. After 4 hours at 40 °C, the suspension was allowed to cool down for an hour whilst being stirred before the particles and capsules were analyzed.

### 2.4 Materials

Each chemical was used without dilution or preconditioning. Melamine formaldehyde resin (MR) was provided by Allnex, marketed under the trade name SETAMINE US-132 BB-71. The epoxy resin (ER) and crosslinker (EC) with the name Araldite Rapid were purchased from Araldite. Dichloromethane (DCM), dinonylnaphthalenedisulfonic acid solution (DNNDSA), and Tween 80 were purchased from Sigma Aldrich. Glycerol (G) was purchased from Laboratoriumdiscounter, and water (W) was collected from the Milli-Q system. PEEK tubing was purchased from Fisher Scientific.

### 2.5 Analysis

**Fig. 3:**
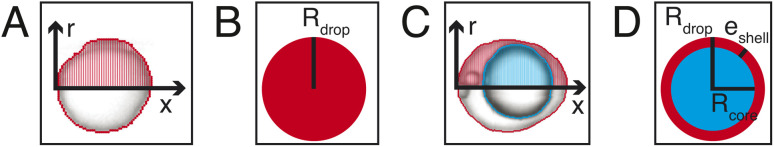
Contours plotted over single emulsion in red (A) and double emulsion (C) droplets (shell: red, core: blue). The encapsulated satellites in capsules were not detected in the analysis. A sketch defining the radius of single emulsion (B) and double emulsion (D) droplets.

The analysis is divided into two parts: on-chip droplet sizing and cured droplet characterization. On-chip sizing is performed by analyzing the images captured by the high-speed camera in an image processing algorithm programmed in MATLAB®. A background image was first subtracted from each frame and the resulting images were binarized to identify the droplets. Then assuming cylindrical symmetry, we sliced the droplet along the horizontal axis *x* which passes through the centre-of-mass of the droplet image and is parallel to the outlet channel. This is shown in Fig. 3A where the contours are plotted overlaying the droplet image. For each half, the surface area of the three-dimensional droplet is calculated by 
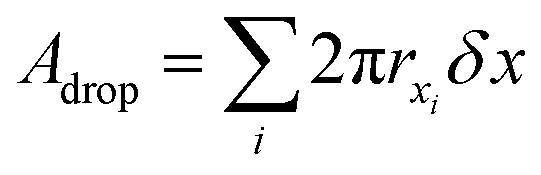
, where *r*(*x*) denotes the distance from the horizontal axis *x* to the droplet edge and *δx* is the width of each slice, and the droplet radius is obtained using 
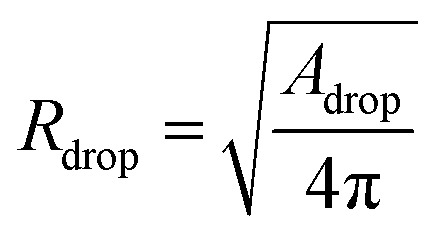
 (see Fig. 3B). The values of *R*_drop_ reported in this paper is the average value of the top and bottom halves. The volume of the drop is then given by 
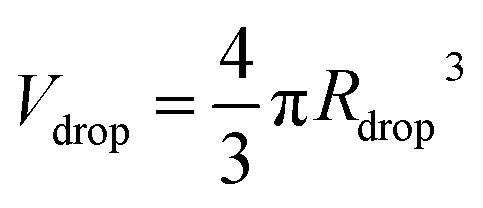
. For the double-emulsion case, the core is segmented by applying an additional image binarization threshold, as shown in Fig. 3C where the contours of the core and droplet are plotted. The core radius *R*_core_ and volume *V*_core_ are found analogously. The shell thickness is then derived from *e*_shell_ = *R*_drop_ − *R*_core_. A sketch defining each parameter is shown in Fig. 3D. To characterize the cured particles or capsules, the cured samples were first imaged under an optical microscope. The pictures were analyzed by fitting circles using a circular Hough transform (imfindcircles function in MATLAB®) to obtain their size distributions. Furthermore, the surface structure and capsule's core were imaged under a scanning electron microscope (SEM). In preparation for these measurements, the particles and capsules were concentrated by removing the surfactant solution and washed successively with milliQ water. The solution was poured away after each rinse and the sample was air dried after a final rinse with isopropyl alcohol. The particles were then sputtered with gold to enhance sample conductivity for SEM observation.

## 3 Results

### 3.1 Single emulsion droplets

**Fig. 4:**
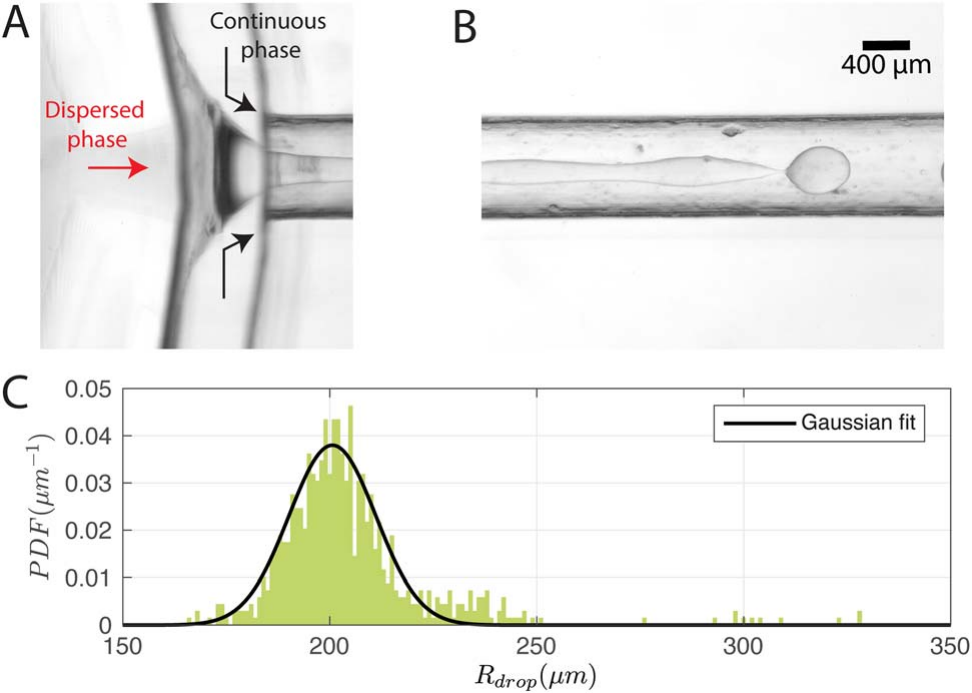
Snapshot of (A) the junction and (B) the jet breaking up inside the chip when producing single-emulsion droplets with (*P*_disp_, *P*_cont_) = (9.5, 77.4) kPa. (C) Measured on-chip droplet size distribution and corresponding Gaussian fit.

Fig. 4A and B show typical snapshots of the chip when single-emulsion droplets are produced with (*P*_disp_, *P*_cont_) = (9.5, 77.4) kPa, where *P* corresponds to the driving pressure of the respective phase; the subscripts disp and cont represent dispersed and continuous phases respectively.

As illustrated, the dispersed phase is hydrodynamically focused into a jet that breaks up into droplets in the outlet channel. A DCM volume of 3.5 ml was chosen to ensure that the jet breaks up before the outlet (distance = 19 mm) at the driving pressures tested. We note that the higher the DCM concentration, the earlier the jet breaks up. The reason is that a lower DCM concentration leads to a higher viscosity, which dampens the growth of the instability thereby delaying jet breakup.^30^ We measured the size distribution of the droplets on the chip and the probability density function (PDF) is plotted in Fig. 4C, together with a Gaussian fit. Note that sometimes much smaller satellite droplets were formed but these were not detected in the on-chip images due to their low contrast. In this configuration, *R*_drop_ = 205 ± 19 μm and the production rate was 110 droplets per s.

**Fig. 5:**
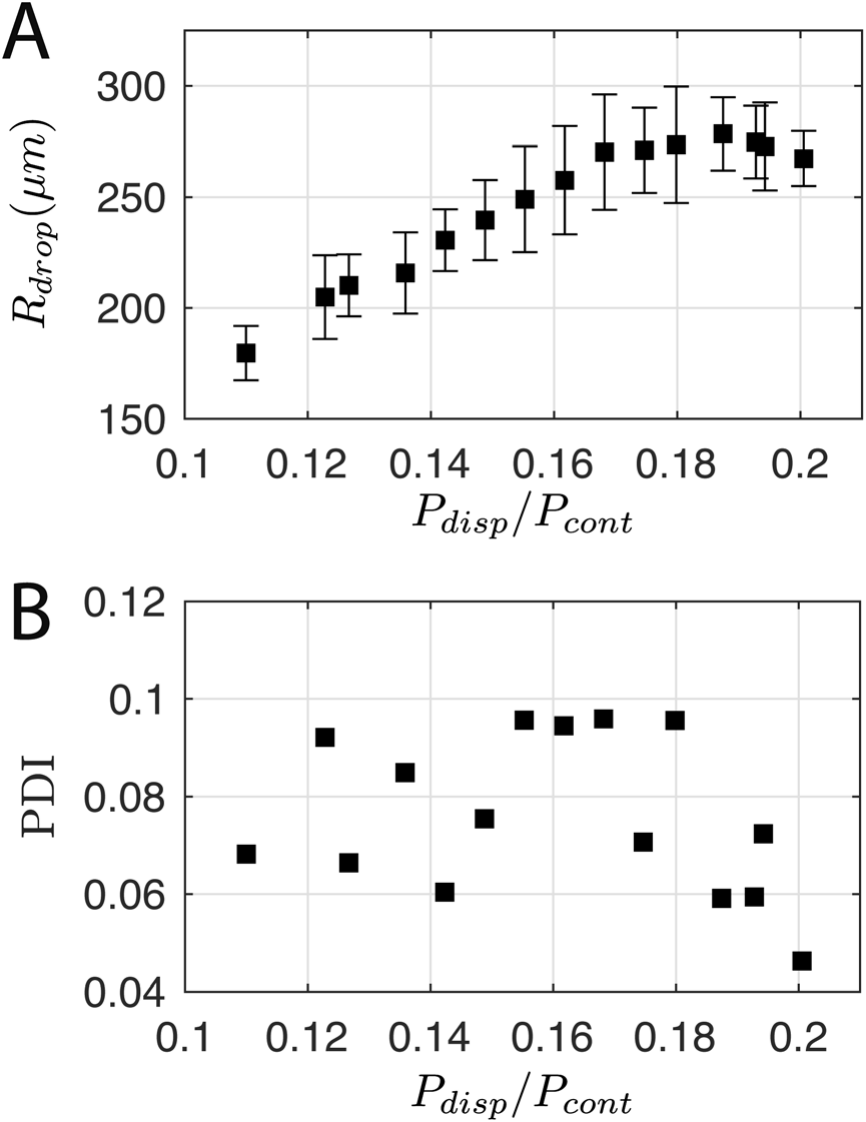
(A) Droplet radius and (B) polydispersity index over a range of dispersed phase pressures *P*_disp_. *P*_cont_ was constant at 77.3 ± 0.1 kPa.

For a first impression of the range of droplets that the chip can produce, *P*_cont_ was fixed at 77.3 ± 0.1 kPa and *P*_disp_ was varied from 8.5 kPa to 15.5 kPa. Plotting *R*_drop_ against *P*_disp_/*P*_cont_ in Fig. 5A indicates that the chip is able to produce droplets with a size range from 180 μm up to 278 μm in radius for the tested values of *P*_cont_. In general, *R*_drop_ increases linearly with increasing *P*_disp_. Assuming that the driving pressure *P* is directly proportional to the flow rate *q* as in Poiseuille flows, this linear increase is consistent with the theory of Guerrero *et al.* (2020):^30^1
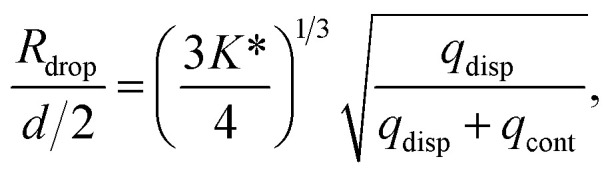
where *d* is the diameter of the dispersed phase channel and K* is a parameter that depends on the fastest growing mode of the Rayleigh–Plateau instability. At the largest values of *P*_disp_, the curve plateaus. In this region, the droplet begins to attain a bullet shape and its width no longer increases with *P*_disp_, presumably because it is now constrained by the presence of the continuous phase. The jet breaks up close to the outlet and its pinch-off location moves back and forth in time. Note that the minimum *R*_drop_ can be further reduced by extending the outlet channel and operating at higher *P*_cont_, at the cost of higher chemical throughput.

The polydispersity index (PDI)—defined as the ratio of the standard deviation to the mean value of *R*_drop_—is shown in Fig. 5B, and ranges from 0.04 to 0.1. For comparison, Donvito *et al.* (2015)^24^ reported a PDI between 0.023 and 0.063 for a 3D-printed T-junction droplet generator. The slightly lower PDI achieved by Donvito *et al.* (2015)^24^ may be due to their operation of the T-junction chip in the squeezing regime with a lower droplet production rate. The squeezing regime in a T-junction chip^31^ is comparable to the dripping regime in a flow-focusing junction,^17^ where the droplet has a low production rate and a diameter comparable to the channel width, hence a lower PDI, *cf.* higher monodispersity.

### 3.2 Particles collection

**Fig. 6:**
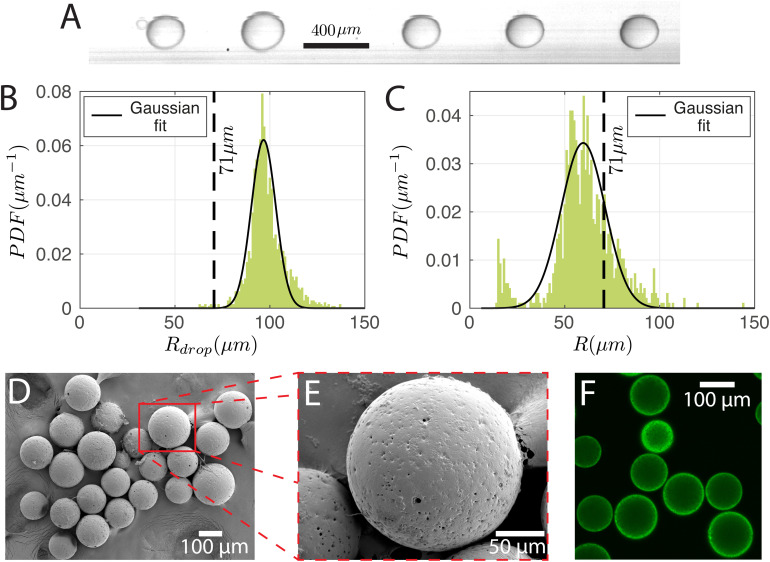
(A) Image of the droplets in the chip before curing. Size distribution of (B) the droplets in the chip and (C) the cured particles with their Gaussian fits. (D) SEM image of the particles and (E) a zoomed image of a single particle. (F) Fluorescent image taken with confocal microscopy (excitation wavelength: 561 nm; detection wavelengths: 570 nm to 620 nm). The dashed lines in (B and C) indicate the calculated particle size assuming complete solvent evaporation.

To produce particles, the droplets generated have to be cured. For this purpose, the dispersed phase mixture was kept the same as in Section 3.1 and the droplets were produced at (*P*_disp_, *P*_cont_) = (13.9, 77.4) kPa. The production rate was 350 droplets per s. A snapshot of the droplets in the chip and the corresponding size distribution are shown in Fig. 6A and B, respectively. *R*_drop_ was 98 ± 9 μm. Cured droplets are observed under SEM demonstrating mono-sizes with spherical shapes as can be seen in Fig. 6D which shows their population and Fig. 6E shows a zoomed image of a single particle. The particles are also imaged with a confocal microscope to demonstrate their ability to fluoresce (Fig. 6F). Fig. 6C displays the size distribution of the cured particles (with a cut-off radius of 14 μm applied for circle detection). The particle radius was 60 ± 17 μm. With *R*_drop_ = 98 ± 9 μm and a solvent volumetric concentration of 63% (59% DCM and 4% BU), the average particle radius *R* would have been reduced to 71 μm upon complete solvent evaporation. The difference between the estimated and observed sizes may be due to variation of the droplet radius over time (Fig. 6B is from a 0.9 s recording while Fig. 6C is from a sample of droplets collected over ∼3 min) and uncertainties in on-chip sizing. We note that there is a secondary peak at small particle sizes in Fig. 6C. This is due to satellite droplets (see leftmost droplet of Fig. 6A) which were not evaluated during on-chip sizing.

### 3.3 Double emulsion droplets

**Fig. 7:**
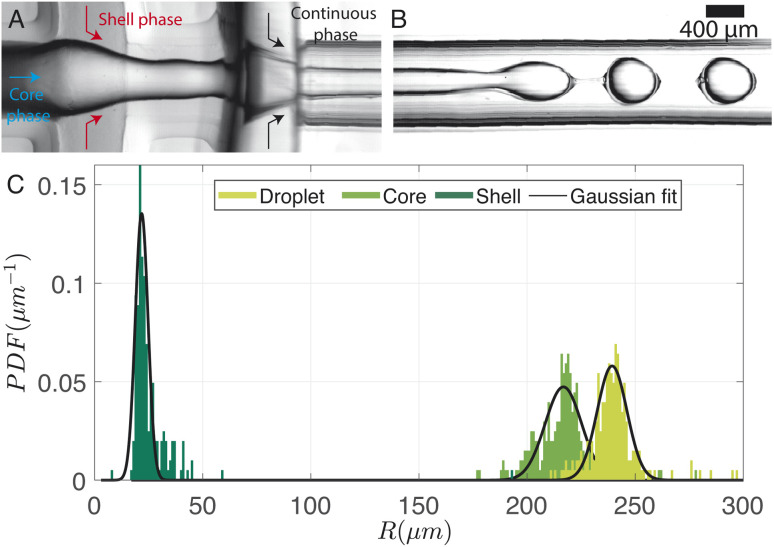
(A) Image showing the three-phase junction with the introduction of continuous, shell (DCM volumetric concentration = 51%), and dispersed phases. (B) Snapshot illustrating the resulting coaxial jet breaking into encapsulated droplets. (C) Size distribution of the droplet radius, core radius, and shell thickness of the samples depicted in (A and B).

A typical image of the double flow-focusing junction with all three phases present is shown in Fig. 7A. In the outlet channel, the coaxial jet pinches off into double-emulsion droplets, as shown in Fig. 7B. The volume of dichloromethane was reduced to 2.5 ml, 1 ml lower the single emulsion droplet. With a volume of 2.42 ml of dissolved material, this results in a DCM-to-solution ratio of 51% (V/V). At this concentration, the shell phase forms a stable jet that does not destabilize between the first and second co-flows. On the other hand, it does break up due to the Rayleigh-Pleateau instability of the second coflow, forming double emulsion droplets. In the case depicted, 625 droplets per s are generated under driving pressures of 75.4 kPa, 60.3 kPa, and 69.9 kPa for the continuous phase (*P*_cont_), shell phase (*P*_shell_), and core phase (*P*_core_), respectively. The subscripts shell and core represent shell and core phases, respectively. Note that the production rate typically varies with droplet size and can be increased by operating the chip at higher pressures. We chose to operate at these pressures to conserve chemicals since it takes ≈30 min to empty the 1 litre container for the continuous phase. The size distributions of the droplets, cores, and shells are shown in the bar plots of Fig. 7C. For this case, *R*_drop_ = 240 ± 13 μm, *R*_core_ = 214 ± 18 μm, and *e*_shell_ = 25 ± 13 μm. The variations in droplet size is due to pressure fluctuations, which slightly changes the length of the jet.

**Fig. 8:**
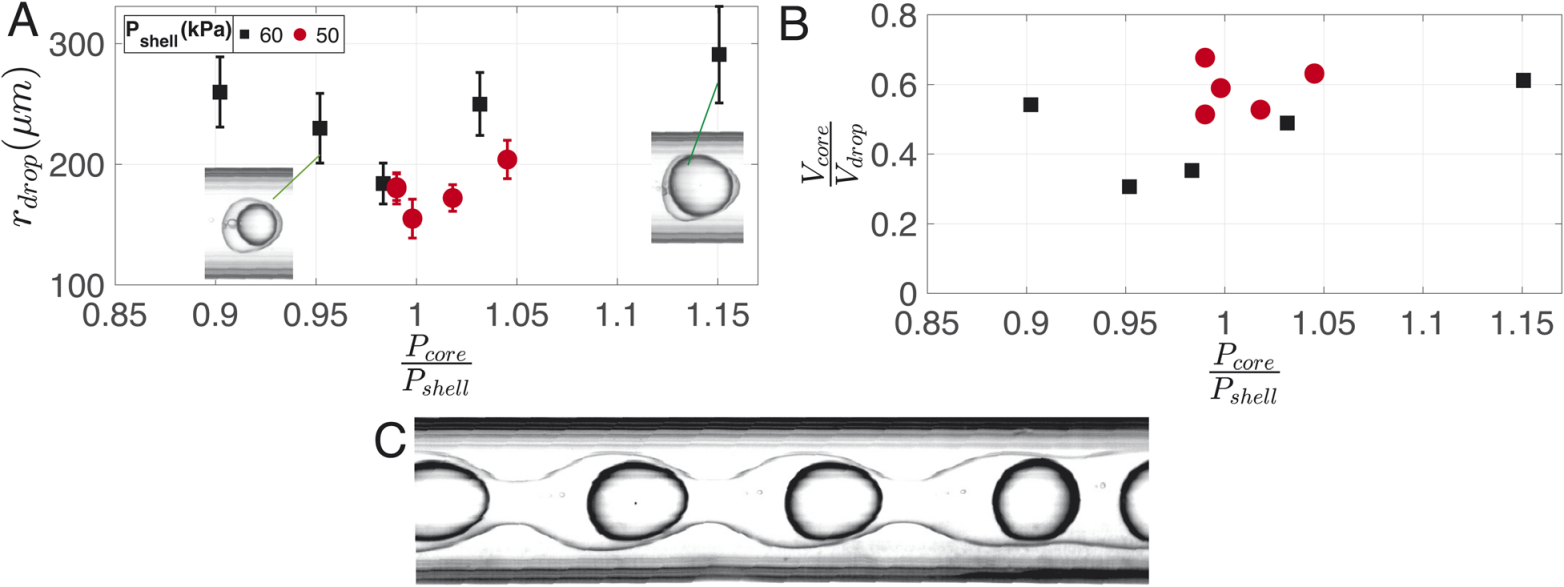
Variation of (A) doplet size and (B) core-to-droplet volume fraction due to changes in core and shell phase pressures indicated in the legend. The marker colors correspond to different shell phase pressures. (C) Coaxial jet behavior above the maximum operating shell phase pressure, where core droplets are formed, but the coaxial jet does not break up inside the channel. *P*_cont_ = 75.4 kPa is kept constant.

Fig. 8A illustrates the variation in droplet size for a constant continuous phase pressure of *P*_cont_ = 75.4 ± 0.2 kPa, while changing the ratio of core to shell phase pressures. The resulting change in core-to-droplet volume fraction is shown in Fig. 8B. The colors of the markers in the plot correspond to different shell phase pressures. At *P*_shell_ = 60 kPa, a stable production regime is observed between 0.9 ≤ *P*_core_/*P*_shell_ ≤ 1.15. In this range, increasing *P*_core_ increases the droplet sizes from 260 μm to 290 μm. Consequently, the core-to-droplet volume fraction increases from 0.5 to 0.6. Expectedly, increasing the pressure of the dispersed phase increases both the overall size and core volume. However, when (*P*_core_/*P*_shell_) ≈ 1, both the overall size and the volume fraction reached a minimum corresponding to 184 μm and 0.35 respectively. The trend continues at a reduced operating pressure of shell phase *P*_shell_ = 50 kPa (red circles). Further investigations into the pressure drop across the junctions while producing droplets would provide additional insights into this phenomenon, but it is beyond the scope of the current study. Below a certain minimum operating pressure ratio, there is insufficient inertia in the dispersed phase to sustain the flow resulting in irregular sizes and, occasionally, a separation of the shell and core phases. Above a maximum operating pressure ratio, core droplets are formed, but the coaxial jet does not break up within the channel (Fig. 8C).

### 3.4 Capsules collection

**Fig. 9:**
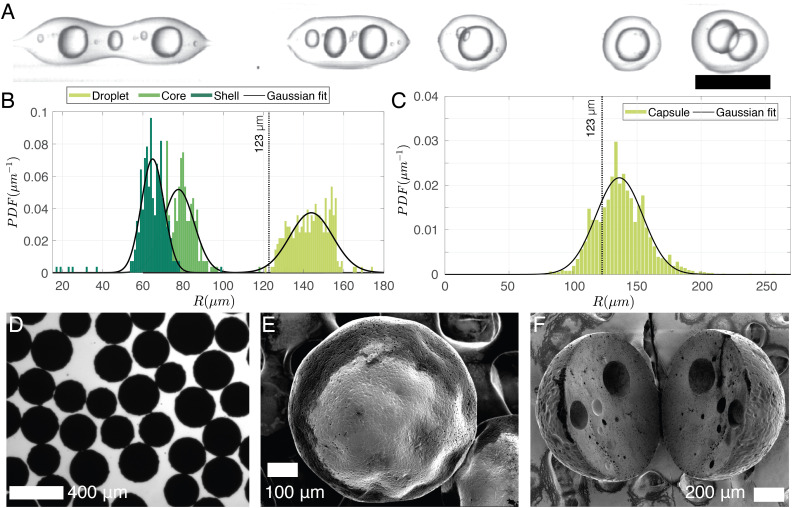
(A) Illustration of a typical jet breakup with a DCM volume concentration of 38% and small core volume (*R*_core_ 79 ± 10 μm). (B) On-chip size distribution of the uncured droplets in (A), and (C) optical sizing of the cured droplets corresponding to the case depicted in (A). (D) A sample of the cured capsules under an optical microscope. (E) Scanning electron microscope image of a capsule and (F) sliced capsules showing the encapsulation of multiple cores. The dotted lines in (B and C) indicate the estimated capsule size assuming complete solvent evaporation.

To keep the core liquid entrapped in the shell during the solidification of the capsules, rapid cross-linking and fast solvent evaporation is required. To achieve this, a preheating coil is utilized to initiate the curing reaction before the sample is collected in the beaker. As the polymer cures it facilitates polymer and solvent separation. Additionally, to accelerate solvent evaporation the volume of DCM is decreased to 1.5 ml, 2 ml lower than the amount of DCM used for particles. It results in a DCM-to-solution ratio of 38% (V/V). This reduction directly influences droplet production, as is evidenced in Fig. 9A, where the increased viscosity of the shell phase leads to the formation of double emulsion with multiple cores at (*P*_cont_, *P*_core_, *P*_shell_) = (77.4, 60.1, 77.6) kPa. As a rough estimate, 50% of the droplet population contains double emulsion droplets. The on-chip droplet size distribution (limited to double emulsions) is plotted in Fig. 9B and shows that the mean core radius (79 ± 10 μm) is notably smaller than in Fig. 7 where a higher concentration of DCM was used, while the overall droplet size and shell thickness are 143 ± 10 μm and 64 ± 8 μm, respectively. Note that higher-order emulsions can be eliminated by increasing *P*_core_/*P*_shell_, although the range of achievable shell thicknesses remains limited as the increased shell phase viscosity makes it challenging for the jet to break up within the channel. Nonetheless, a smaller core (thicker shell) has the advantage of minimizing the likelihood of the core escaping due to liquid shell rupture during curing. After the capsules are cured, they are imaged under an optical microscope (Fig. 9D), and their size distribution is shown in Fig. 9C. Assuming that all solvent in the shell phase (38% DCM and 6% BU totaling 44% solvent-to-solution ratio (V/V)) evaporates, the expected capsule size is 123 μm (dotted line in Fig. 9(B and C)). However, if the shell ruptures and the core dissolves in the continuous phase, the resulting particle size would be around 111 μm. Considering a 10% error in optical sizing, these estimates are quite close to the measured size of 138 ± 21 μm. A scanning electron microscope image of a typical capsule with a radius of ∼200 μm is shown in Fig. 9E. It is larger than our predicted size (123 μm) and is likely to have an encapsulated core. Within the same size range, Fig. 9F displays a sliced capsule demonstrating multiple encapsulated cores that are most likely as a result of the curing of a double of higher-order core droplet.

## 4 Discussion

**Fig. 10:**
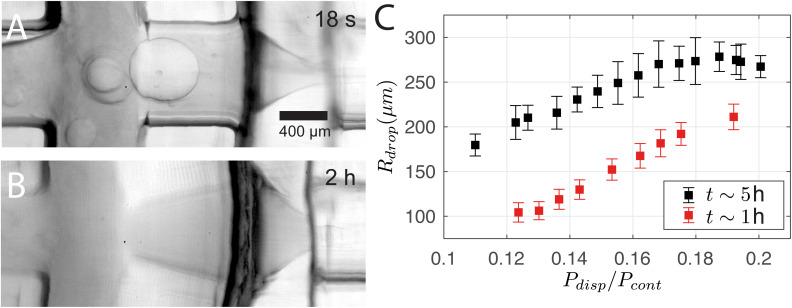
(A and B) Deformation of the junction region of the chip that deforms under action of direct contact with the shell phase. (C) Single-emulsion droplet radius *R*_drop_ at different *P*_disp_/*P*_cont_ generated at different times *t* of 1 hour and 5 hours of operation.

3D printing is advantageous for its fast and flexible production cycle and high chemical resistance with several organic mediums. However, channels that are in direct contact with dichloromethane for an extended period of time lead to deformation. The use of DCM in this study is driven by several key factors. First, DCM exhibits negligible solubility in the continuous phase, which prevents unwanted mixing and promotes effective droplet formation. Additionally, it is compatible with the resin employed in the system. Its density is higher than that of the bulk phase, which aids in maintaining suspension stability during stirring, unlike toluene, which leads to system collapse. Lastly, the low boiling point of DCM helps prevent the dissolution of the core material into the surrounding medium, a problem that arises when the solvent temperature approaches the core's boiling point. Fig. 10A and B show that the channel between the two junctions deforms from a cylindrical to a diverging profile over time. Note that it returns to the original cylindrical profile in ∼12 h of inactivity. The deformation changes the operating regime. For instance, the size of the droplets increases with time as shown in Fig. 10C, where the single-emulsion droplet radius *R*_drop_ is plotted against *P*_disp_/*P*_cont_ while being measured at two time points *t* at 1 hour and 5 hours of operation. We stress that the difference in *R*_drop_ cannot be attributed to fluctuations in *P*_cont_ as *P*_cout_ was 0.4 kPa higher at *t* ∼ 5 h. This deformation also explains the measurable change of *R*_drop_ between Sections 3.1 and 3.2 despite the similar control parameters. For the droplet cases presented in Sections 3.1 and 3.3, the chip was deformed for more than 1 hour of operation before the experiments started.

## 5 Conclusion

A three-dimensional millifluidic chip design with two flow-focusing junctions that can be easily manufactured using a 3D printer is presented. The chip can produce both single and double-emulsion microdroplets depending on the inlet configuration. Droplet production occurs by coflow, which causes jet breakup *via* a Rayleigh–Plateau instability. Using a mixture of dichloromethane, epoxy resin, hardener, melamine crosslinker, and dinonylnaphthalenedisulfonic acid, the chip produces monodispersed droplets with sizes ∼ *O* (100) μm with a polydispersity index between 0.04 and 0.1. Operating at low driving pressures, we measured that the production rate in the jetting regime is around 100 Hz and 600 Hz for single and double emulsion droplets, respectively. By varying the core phase driving pressure, a large range of droplet sizes and shell thicknesses can be obtained. The overall size for single emulsion droplets increases with increasing dispersed phase pressure. In the case of double emulsion droplets, the droplet size reaches a minimum before increasing with increasing core phase pressure, while a constant shell phase pressure is maintained. By heating the suspended droplets at 40 °C for 4 hours, the droplets were cured into either particles or capsules. This demonstrates the viability of using 3D-printed chips in handling complex curable mixtures.

Still, multiple questions remain to be addressed. In our experiments, we drove the system at low pressures to conserve chemicals at the expense of restricting the accessible phase space. Future studies can increase the range of driving pressures which would reveal the full range of particle sizes that can be achieved by the chip. Furthermore, while our investigation shows the possibility of generating particles and capsules using one model system, it will be practically useful to examine the compatibility of the 3D-printed chip for other systems and the monodispersity of the end products in order to assess the versatility of the 3D-printed chip.

## Data availability

Data for Fig. 4(C), 5(A, B), 6(B, C), 7(C), 8(A, B), 9(B, C) and 10(C) in “Additive manufacturing of 3D flow-focusing millifluidics for the production of curable microdroplets”, is available online in the “4TU.ResearchData” repository with the DOI: https://data.4tu.nl/datasets/bee2260c-630d-46cb-aa1d-a10032fa8a8f.

## Conflicts of interest

There are no conflicts to declare.
